# *In vitro* and *in vivo* properties of a fully human IgG1 monoclonal antibody that combats multidrug resistant *Pseudomonas aeruginosa*

**DOI:** 10.3892/ijmm.2012.1040

**Published:** 2012-06-20

**Authors:** AZMI ADAWI, CARLO BISIGNANO, TIZIANA GENOVESE, ANGELA FILOCAMO, CAMELLIA KHOURI-ASSI, ANAT NEVILLE, GIORA Z. FEUERSTEIN, SALVATORE CUZZOCREA, LEWIS F. NEVILLE

**Affiliations:** 1 Lostam BioPharmaceuticals, Nazareth, Israel;; 2 Department of Pharmacobiology, School of Pharmacy and; 3 Department of Clinical and Experimental Medicine and Pharmacology, School of Medicine, University of Messina, Messina, Italy;; 4 St. Vincent Hospital, Nazareth;; 5 Department of Family Medicine, Ben-Gurion University of the Negev, Beer-Sheva, Israel;; 6 Farmacon Consulting, Bryn Mawr, PA, USA

**Keywords:** *Pseudomonas aeruginosa*, multidrug resistance, monoclonal antibody, passive immunization, flagellin

## Abstract

The development of an anti-bacterial drug in the form of a monoclonal antibody (mAb) targeting an exposed virulence factor, represents an innovative therapeutic strategy. Consequently, a fully human IgG1 mAb (LST-007) targeting *Pseudomonas aeruginosa* (PA) flagellin type b was recombinantly expressed and characterized *in vitro* and in an infection model driven by a multidrug resistant (MDR) PA strain. LST-007 demonstrated a highly specific binding towards whole PA bacteria harboring flagellin type b and its recombinant counterpart, with a K_D_ of 7.4×10^−10^ M. In bioactivity assays, LST-007 or titers of Cmax sera derived from pharmacokinetic studies, markedly attenuated PA motility in an equipotent manner. *In vivo*, parenteral LST-007 (20 mg/kg) given as a single or double-dosing paradigm post-infection, afforded survival (up to 75% at Day 7) in a lethal model of pneumonia driven by the intratracheal (i.t.) instillation of an LD_80_ of the MDR PA isolate. This protective effect was markedly superior to that of imipenem (30% survival at Day 7) and totally devoid with an irrelevant, human isotype mAb. These data lay credence that LST-007 may be a valuable adjunct to the limited list of anti-bacterials that can tackle MDR PA strains, thereby warranting its continued development for eventual clinical evaluation.

## Introduction

Regrettably, the emergence of multidrug resistant (MDR) strains of bacteria during the last 2–3 decades has failed to ignite intensive research and development efforts towards the discovery of novel antimicrobial drugs. This is exemplified by the fact that only 2 new classes of antibiotics have been marketed since 1970 (1,2). The Gram-negative bacterium *Pseudomonas aeruginosa* (PA) is an opportunistic pathogen and may be regarded as a nosocomial ‘predator’ especially in immunocompromised patients (3,4). PA infections may cause life-threatening conditions such as ventilator-associated and hospital-acquired pneumonias as well as bloodstream and urinary tract infections in susceptible patients (5,6). Additionally, PA plagues cystic fibrosis (CF) patients with at least 50% of the ∼60,000 population in the US and EU permanently colonized with the bacterium (7,8). Indeed, PA is a major cause of morbidity and mortality in these patients (7,9). A remarkable feature of PA lies not only in its genomic diversity with a plethora of ORFs capable of degrading antibiotics (10) but also its capacity to acquire external genomic elements providing additional resistance mechanisms (11). Ultimately, MDR PA can be a grave problem, especially in the critical care setting where mortality rates for certain infections may range from 38 to >70% (12). Thus, designing the appropriate antimicrobial therapy may be a clinical dilemma due to the paucity of effective and safe drugs that can combat MDR strains of PA.

An alternative and highly innovative anti-bacterial therapeutic approach apart from classical antibiotics is to disarm bacterial pathogens thereby augmenting the host’s innate immune system to clear the infection (13). The notion of disarmament of bacterial pathogens by targeting virulence factors has allowed a resurgence in the importance and potential therapeutic role of anti-infective monoclonal antibodies (mAbs). Such advances have been catalyzed by a number of methodological breakthroughs which have permitted not only the rapid generation of fully human or humanized mAb candidates but also the capabilities to recombinantly express mammalian cell-derived mAbs at g/l quantities (14,15). Pre-clinically, a number of pharmacological studies have demonstrated the effectiveness of anti-PA virulence mAbs which have justified their clinical evaluation (16–18). For example, KB001 and panobacumab, human mAbs targeting PA virulence factors PcrV and 011 LPS serotype, respectively, have demonstrated positive clinical findings, albeit in a limited number of hospital-associated pneumonia and CF cases when added to standard-of-care treatment (19,20). These findings support passive immunization approaches targeting virulence factors especially since from an evolutionary point of view, they may be refractory to selection pressures due to their pivotal roles in bacterial infectivity and survival.

In accord with the above, we also believe that passive immunization with anti-PA virulence mAbs is a viable therapeutic proposition, representing an unfulfilled medical requirement. In contrast to the PcrV and 011 LPS virulence factors, our preferred PA target is the surface-expressed, single polar flagellum of which flagellin is the primary protein component (21,22). Flagellar structures are pivotal for a number of PA’s functions including bacterial motility, attachment and invasion to susceptible cells as well as being highly pro-inflammatory via the Toll-like receptor 5 (TLR5) (21–23). Consequently, a neutralizing mAb targeting the flagella of PA may intervene at a variety of critically important steps and curtail the catastrophic sequelae of events that lead to end-organ infection, dysregulated inflammation and mortality.

It should be noted that in PA, 2 types of flagellin have been identified and termed type a and type b which may be discriminated on the basis of molecular size and reactions with type-specific polyclonal and mAbs (24,25). In contrast to *salmonella* flagellins, PA flagellin types a and b do not exhibit phase variation since a single strain produces a single type of flagellin with no switching between types a and b (26). Numerous *in vivo* studies have pointed to the protective effects of either polyclonal antibodies or specific anti-flagellin mAbs following infection with antibiotic-sensitive PA strains harboring the appropriate, homologous flagellin type (27–31). Indeed, such antibody preparations provided equivalent protection to imipenem, a standard-of-care carbapenem antibiotic (30,31). Since >98% PA strains harbor a phenotypic type a or b flagellin (24,27), an appropriate mAb therapy governed by *a priori* knowledge of the infecting flagellin type may permit a ‘patient-tailored’ anti-infective therapy and thus a higher probability of clinical success.

In the present study, we applied a battery of *in vitro* and *in vivo* tests to characterize our fully human, lead mAb termed LST-007 that targets PA flagellin type b. To our knowledge, this is the first report demonstrating that an anti-infective PA mAb administered as a monotherapy, can curb lethality driven by a clinically relevant MDR PA strain.

## Materials and methods

### Reagents

All general chemicals were purchased from Sigma (Rehovot, Israel) and HyLabs (Rehovot, Israel). Imipenem in the form of the marketed drug Tienam, was acquired from the Department of Pharmacy, University of Messina. Bacterial PA strains 27853, 25619 and 9721 were purchased from Microbiologics (USA). Ka02, a hospital-derived MDR PA strain, demonstrated sensitivity only to amikacin, colistin and partial sensitivity to gentamicin as determined from Vitek screens. LST-003, a proprietary chimeric mAb targeting PA flagellin type a was transiently expressed in CHO-K1 cells and used in certain *in vitro* studies. A human IgG1 isotype control mAb (32) was used in a number of *in vitro* and *in vivo* studies.

### Expression and purification of recombinant PA flagellin type b

The fliC gene (Pa1092 from the PA genome database) corresponding to flagellin type b of the laboratory strain PAO1, was used as the template for recombinant expression. This gene sequence was custom synthesized to permit appropriate codon usage for optimal expression in *E. coli* without modifying its primary amino acid sequence (Geneart, Germany). This cDNA sequence was engineered with 5′ *Nde*I and 3′ *Hind*III restriction sites enabling direct ligation into a pET28a expression plasmid cleaved with these respective enzymes. The resultant recombinant protein harbored an N’-terminal histidine-6 tag enabling immobilized metal affinity chromatography (IMAC) purification. The pET28a/ flagellin type b recombinant plasmid was transformed into BL21 DE3 STAR cells (Invitrogen, Carlsbad, CA, USA) and a single colony was grown in 10 ml LB containing 50 μg/ml kanamycin overnight. Thereafter, 0.2 ml of the overnight culture was taken to inoculate 50 ml pre-warmed LB-kanamycin and at an OD of 0.3, IPTG was added to a final concentration of 1 mM to induce expression for 3 h at 37°C. Since the bulk of the recombinant protein was shown to reside in the insoluble fraction, induced bacterial pellets were resuspended in 50 mM phosphate buffer (pH 7.4) containing IGEPAL, sonicated and following centrifugation, the insoluble pellet was taken for IMAC as follows. The pellet was solubilized in buffer containing 6 M GuHCl and subsequently purified by IMAC (HiTrap chelating 5 ml column charged with nickel). Recombinant PA flagellin type b was refolded on the column using a gradient 6-0 M GuHCl and an elution was performed using an imidazole gradient (0–500 mM). Peak fractions were pooled, dialyzed against PBS, quantified using BCA and adjusted with PBS to a stock concentration of 1 mg/ ml and stored at −20°C. Coomassie gel staining demonstrated that the Mw of PA flagellin type b was ∼55 kDa with >95% purity. In small-scale expression and purification studies, recombinant PA flagellin type a was produced in an identical manner as described for flagellin type b and yielded a protein of Mw ∼45 kDa with >95% purity.

### Expression and purification of LST-007

LST-007 was isolated from EBV-transformed human B cells (unpublished data) and its respective V_H_ and V_L_ cDNA chains were taken for expression using a commercially available, platform technology. In brief, following cloning and expansion of transduced LST-007 V_H_ and LST-007 V_L_ pools, shaker flasks of CHO cells supported in serum-free media were co-infected and clarified supernatant were taken for standard purification using a MabSelect medium. Purified LST-007 was concentrated in a stirred cell, dialyzed vs. phosphate buffer and quantitated at 280 nm. Bioburden of sterile-filtered material was 0 cfu/ml with levels of endotoxin <0.04 EU/mg. Final concentration of LST-007 was quantified at 13.15 mg/ml and stored as a number of 1-ml aliquots at −20 and −80°C.

### Binding of LST-007 towards recombinant PA flagellin type b in ELISA

Wells of Maxisorp ELISA plates (#442404; Nunc Brand Products) were coated with 250 ng of recombinant PA flagellin type b and incubated with orbital shaking for 2 h at room temperature. Thereafter, unbound antigen was removed and 200 μl blocking solution comprised of PBS-10% fetal bovine serum (FBS; #04-001-1A; Biological Industries) was added overnight at 4°C. Following removal of blocking buffer, 50 μl LST-007 in blocking solution was added over a range of 0–208 pM (0–31.25 ng/ml) and allowed to incubate for 2 h at room temperature. LST-007 was decanted and the wells were washed 3 times with 200 μl of PBS containing 0.05% Tween-20. A secondary antibody consisting of a 1:10,000 dilution of goat anti-human IgG-Fc-HRP conjugate (#A80-104P; Bethyl) in blocking solution was added for 1 h at room temperature. Following identical washing as described above, 50 μl of TMB solution (#ES001; Millipore) was added after which the colorimetric reaction was quenched following the addition of 50 μl 10% H_2_SO_4_. The plates were read in an ELISA reader at OD 450 nm. Blocking buffer with the sole presence of primary or secondary antibodies was used as blank controls.

### Binding of LST-007 towards immobilized, whole PA bacteria in ELISA

PA strains were grown in 5 ml LB medium at 37°C in an orbital shaker for 18 h, pelleted and washed twice with PBS. Following resuspension of the bacterial pellet with a small volume of PBS, the volume was adjusted to obtain an OD 600 nm of 0.2. To permit bacterial binding, 50 μl of poly-L-lysine (PLL) of a 1 μg/ml solution was added to the wells of Maxisorp ELISA plates and incubated for 30 min at room temperature with orbital shaking. Following removal of PLL, 50 μl of the PA suspensions were added to plates and immediately centrifuged at 1,500 rpm for 20 min. Thereafter, supernatants were carefully removed with a multi-channel pipettor and adsorbed PA bacteria were irreversibly fixed to wells by adding 75 μl 0.2% formaldehyde for 15 min at room temperature. Following removal of formaldehyde and brief drying, plates were blocked overnight at 4°C with 200 μl PBS-10% FBS. The blocking solution was removed and 50 μl of 0.5 μg/ml LST-007, LST-003 or human isotype control mAb was added for 60 min at 37°C. The ELISA was continued in an identical manner with recombinant PA flagellin type b as described above.

### Isoelectric focusing (IEF) studies

IEF gels (5% T/3% C; 0.25 mm thick) were prepared by polymerization of acrylamide and N,N’-methylenebisacrylamide on GelBond^®^ PAG film (124×258 mm), followed by washing, drying, and reconstitution with 2% Pharmalyte 3–10, 10 mM glutamic acid, 10 mM lysine, and 32% (v/v) glycerol. IEF was performed on a horizontal unit, at 15°C, under a nitrogen atmosphere, 10 cm between electrodes. Gels were prefocused for 500 V-h (500 V, 60 min). Samples were loaded as drops on the gel surface and focused for 9,500 V-h (500 V, 30 min; 1,000 V, 30 min; 2,000 V, 30 min; 3,500 V, 133 min). Gels were fixed in 20% (w/v) trichloroacetic acid and stained with Coomassie G-250 using the colloidal methodology (33).

### Surface plasmon resonance (SPR) studies

SPR was performed on a Biacore 3000 instrument (Biacore, Uppsala, Sweden). Recombinant PA flagellin type b was diluted in 100 mM sodium acetate (pH 4.6) to a final concentration of 50 μg/ml and immobilized on a CM5 Biacore sensor chip. The antigen was then streamed over the sensor chip for 5 min at a rate of 10 ml/min. The binding assay was performed by injecting LST-007 at 16 different concentrations ranging from 0.0 to 50 nM at a flow rate of 20 ml/min at 25°C. These conditions resulted in a linear relation between the concentration of LST-007 and the maximal (steady-state) response, indicating the pseudo first-order regime in relation to the immobilized ligand. The net signal was obtained by subtracting the blank signal (dextran matrix). The association phase for LST-007 binding was monitored for 4 min, while the dissociation phase for 3 min. Responses were monitored as a function of time by generating the traces (sensorgrams) at 25°C. Multi-concentration data were globally fitted using the BIAevaluation 3.2 software supplied by Biacore to calculate affinity constant of LST-007.

### In vitro PA motility assays

Motility studies were performed as previously described (34). In brief, freshly prepared PA colonies were taken for motility experiments as follows. Soft liquid agar was prepared by autoclaving LB media containing 1% tryptone, 0.5% NaCl, 0.3% yeast extract and 0.3% soft agar. This solution was transferred to a water bath at 40°C for 30 min. After 30 min, the solution was aliquoted into working volumes and mAbs or diluted Cmax sera were added at the desired concentrations. In some assays, 10 cm plates were used whereby 20 ml of the mAb-containing soft agar was poured. In some assays, 24-well plates were used, with 1 ml of the mAb-containing soft agar poured/well. Plates were allowed to solidify for ∼30 min at room temperature within a bacterial culture hood and wells were inoculated with the desired freshly prepared PA strain by picking a colony with a sterile toothpick and carefully stabbing a few mm into the mAb-impregnated agar. Plates were wrapped in parafilm to prevent dehydration and incubated at 30°C for 12–16 h to maximize flagellin expression. Wells were photographed and the motility was measured as the diameter of the halo phenotype that surrounds the central bacterial growth.

### LST-007 pharmacokinetic studies

Female CD-1 mice, age 10–12 weeks (20–25 g) were used. Animal handling was performed according to the National Institute of Health (NIH) and the Association for Assessment and Accreditation of Laboratory Animal Care (AAALAC). During acclimation (5 days) and following LST-007 dosing, mice were housed in a specific pathogen-free environment with 3 mice/cage, in polypropylene cages fitted with solid bottoms and filled with autoclaved sawdust as bedding material. Animals were provided *ad libitum* with a commercial rodent diet and free access to autoclaved drinking water supplied to each cage. Automatically controlled environment conditions were set to maintain a temperature of 22–25°C with a 12 h light/12 h dark cycle and air changes in the study room.

LST-007 was prepared at a concentration of 1 mg/ml and injected at 5 ml/kg to achieve a target dose of 5 mg/kg. A total of 27 mice were taken for simultaneous PK sampling from bleeds and bronchoalveolar lavage (BAL) fluid, with 3 mice/time point sacrificed for both samplings. Sampling time points were 5 min (Cmax), 4, 8, 24, 48, 96, 120, 168 and 240 h. Additionally, 3 mice were sampled at 5 min following the injection of saline at 5 ml/kg. In brief, mice were injected intravenously (i.v.) with LST-007 in the tail vein using a tuberculin syringe and a 30G needle. At the designated time points, mice were anesthetized using an intraperitoneal (i.p.) injection of 85 mg/kg xylazine and 5 mg/kg ketamine and bled, ∼500 μl from the orbital sinus. The blood was collected into 1.5 ml Eppendorf tubes, centrifuged and the upper sera layer was aliquoted and stored at −80°C until required. While the mice were still under anesthesia, they were placed on their backs and the airway was exposed for collection of BAL fluid by connection of a veinflow to the airway attached to a 26G needle and 1 ml syringe. A total volume of 700 μl of saline was used to wash the lungs with return BAL volumes ∼500 μl. Following centrifugation, the clarified BAL supernatant was removed, aliquoted and stored at −80°C until assay.

For sampling LST-007, an ELISA was adapted whereby dilutions of sera (1:500–1:2,000) or BAL fluid (1:100–1:400) were added to ELISA plates coated with a goat anti-human IgG Fc specific antibody. Following incubation and blocking (see above), wells were incubated with a detecting anti-human κ-HRP antibody followed by colorimetry with TMB. Plates were read at 450 nm after quenching with H_2_SO_4_. Since the BAL fluid compartment undergoes ‘dilution’ via flushing with saline, recovered BAL samples were taken for BUN determinations (AML, Herzliya, Israel), enabling normalization. As an example, BUN levels in BAL were measured at 1.67 mg/dl as compared to concentrations of 25 mg/dl in mouse sera. Thus, BAL-derived LST-007 concentrations determined in ELISA were normalized following a 15-fold multiplication.

### Mouse model of pneumonia

Adult C57 mice (25 g; Harlan Nossan, Milan, Italy) were housed in a controlled environment and provided with a standard rodent diet and water. Animal care was in compliance with the Italian regulations for protection of animals used for experimental and other scientific purposes (DM 116192) as well as the EEC regulations (OJ of ECL 358/1 12/18/1986). The mice were housed in cages with filter tops in specific pathogen-free conditions. They were briefly anesthetized with inhaled Sevorane (Abbot Laboratories) in an oxygenated chamber and placed in a supine position with their heads elevated ∼30°. Initially, a limited number of mice were dedicated to the animal surgery and intratracheal (i.t.) procedure by validating 100% animal survival and normal behavior throughout the 7 days post instillation of 50 μl lactated Ringer’s solution into the left lung. Thereafter, a LD_80_ pneumonia model was established at 3 days post-infection, elicited by the i.t. administration of the MDR PA strain Ka02 at 10^6^ cfu in 50 μl lactated Ringer’s solution. Using this LD_80_, the biological activity of LST-007 or control treatments (i.v. 6 ml/kg) on animal survival were investigated in the following 5 experimental groups: group 1 (n=20): i.t. Ka02 followed by i.v. formulation buffer (FB) at +60 min and +25 h after infection; group 2 (n=20): i.t. Ka02 followed by i.v. LST-007 (20 mg/kg) at +60 min and FB at +25 h; termed ‘LST-007x1’; group 3 (n=20): i.t. Ka02 followed by i.v. LST-007 (20 mg/kg) at +60 min and + 25 h (20 mg/kg); termed ‘LST-007x2’; group 4 (n=20): i.t. Ka02 followed by freshly prepared, i.p. imipenem (25 mg/kg; 0.25 ml of a 2.5 mg/ml solution) given b.i.d. for 3 days post-infection at time points of +60 min, +5, +24, +29, +48 and +53 h; group 5 (n=15): i.t. Ka02 followed by i.v. human isotype mAb (20 mg/kg) at +60 min and + 25 h (20 mg/kg). For groups 2, 3 and 5, mAb stock concentrations of 3.33 mg/ml were provided from which 0.15 ml were injected per mouse (i.e. 6 ml/kg).

Statistics were run in Stata version 7 (Stata Corp., College Station, TX, USA). The differences between the antibody or imipenem treated groups vs. FB-treated groups were analyzed. Dichotomous outcomes were compared using Fisher’s exact test and continuous variables by the Student’s t-test. All statistical tests were two-tailed. Differences were considered to be statistically significant with a P-value ≤0.05.

## Results

### In vitro characterization of mammalian-expressed LST-007

**Figure 1.**
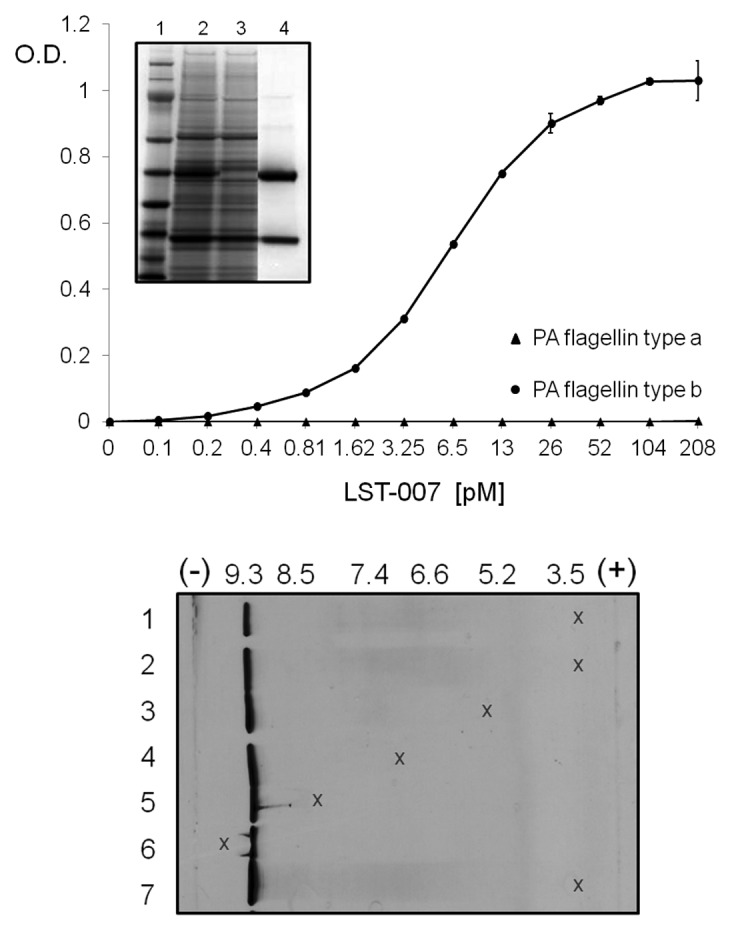
*In vitro* properties of recombinant LST-007 expressed and purified from CHO cells. Upper panel depicts purification profile of LST-007 as shown by Coomassie gel staining of clarified CHO cell supernatant (lane 2), wash fraction (lane 3) and eluted LST-007 product (lane 4), under reducing conditions. Lane 1 is a Mw (kDa) marker of sizes 191, 97, 64, 51, 39, 28, 19 and 14. ELISA curve demonstrates concentration-dependent binding of LST-007 from at least 3 independent experiments towards *E. coli*-expressed, purified PA flagellin type b (circles) with no reactivity towards flagellin type a (triangles). Lower panel demonstrate IEF properties of LST-007. Samples of 2 mg/ml LST-007 were loaded as 5 μl drops to the gel surface at designated positions (denoted by x) at 1 cm (lane 2), 3 cm (lane 3), 5 cm (lane 4), 7 cm (lane 5) and 9 cm (lane 6) from the anode. Additionally, LST-007 was loaded at 1 cm from the anode at a concentration of 1 mg/ml (lane 1) and 5 mg/ml (lane 7). The results show that LST-007 is a very basic protein of pI 9.3.

Recombinant LST-007 was expressed in CHO cells and purified to homogeneity. The upper panel of Fig. 1 shows the Coomassie gel staining of an SDS-PAGE loaded with clarified media containing secreted LST-007 (lane 2), a washed fraction (lane 3) and 4 μg of the final eluted LST-007 product (lane 4) depicting the heavy and light chains. Lane 1 is a molecular weight (Mw) marker of bands 14, 19, 28, 39, 51, 64, 97 and 191 kDa. LST-007, prepared as a sterile-filtered solution at 13.15 mg/ml, was verified to be free of bacterial burden with LPS <0.04 EU/mg. In ELISA studies employing recombinantly expressed flagellin type b as the immobilized antigen, LST-007 demonstrated a concentration-dependent increase in OD which peaked at 208 pM (=31.25 ng/ml LST-007). No binding was observed whatsoever with PA flagellin type a (Fig. 1). In IEF studies, when loaded at various locations on the IEF gel at different amounts, LST-007 was demonstrated to be a very basic protein with a pI of 9.3 (Fig. 1, lower panel). When added maximally at 25 μg (lane 9), two very light additional bands were observed next to the main band at pH 9.2. While it is possible that the heavy pH 9.3 band consists of several unresolved bands, for the most part, the isoelectric focusing data at pH 9.2 demonstrates a high level of LST-007 electrostatic homogeneity. The basic pI of LST-007 is similar to the pIs of a number of marketed mAbs from theoretical and experimental measurements (35).

**Figure 2.**
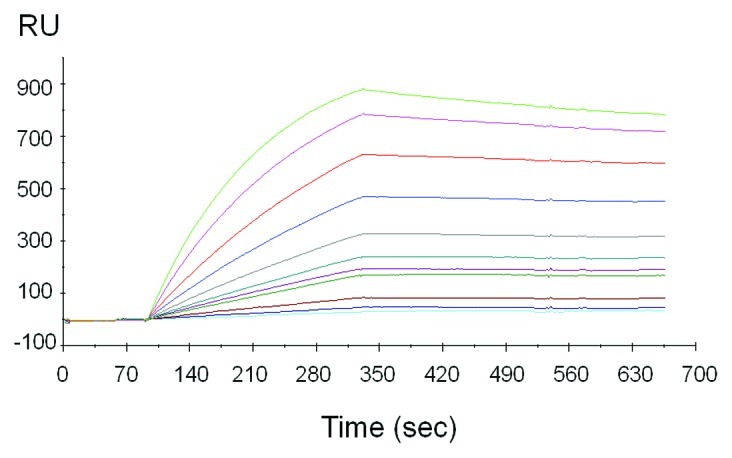
Concentration-dependent, sensorgram traces of LST-007 binding towards purified, *E.coli*-expressed PA flagellin type b by surface plasmon resonance (SPR) using Biacore 3000. Binding of purified LST-007 towards its purified antigen on coated CM5 biosensor chips. Sensorgram traces depict binding of LST-007 at 0.5, 0.75, 1, 2.5, 3, 4, 5, 7.5, 10, 15 and 20 nM with proportional increases in resonance units (RU). The affinity of LST-007 affinity towards recombinant PA flagellin type b was calculated at 7.4×10^−10^ M.

The specificity of the above ELISA data was re-enforced in surface-plasmon resonance (SPR) studies. Here, LST-007 (0.5–20 nM) was streamed over immobilized PA flagellin type b bound to a sensor chip and concentration-dependent increases in the signal were observed as demonstrated in the sensorgrams (Fig. 2). Analysis of these data confirmed the high affinity of LST-007 towards recombinant PA flagellin type b which was calculated at 0.74 nM.

**Figure 3.**
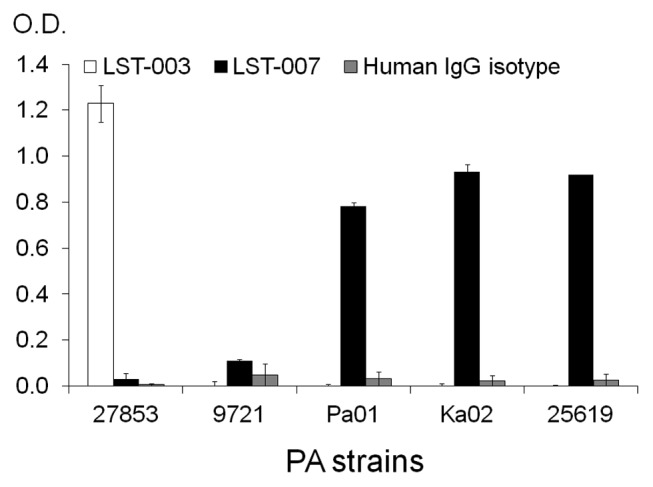
LST-007 demonstrates specific binding towards immobilized whole PA bacteria harboring flagellin type b, but not type a, in fixed ELISA. Binding of LST-007 (0.5 μg/ml) was investigated towards formaldehyde-fixed, intact PA bacterial strains known to express type a (Pa27853) or type b (Pa01, Ka02, 25619) flagellin. Additionally, a non-motile PA strain (9721) was used as a control. LST-007 demonstrated specific binding towards all PA type b flagellin strains with no binding towards Pa27853 or 9721. A proprietary chimeric mAb termed LST-003, which solely binds PA flagellin type a, served as a control in the ELISA screen. No binding to any of the PA strains was observed with a human isotype control mAb at 0.5 μg/ml.

To further scrutinize the specificity of LST-007 binding and assess its capability to bind the ‘naturally-occurring’ flagellin type b, ELISA studies were established whereby the PA target was in the form of immobilized whole PA bacteria tethered to ELISA plates via poly-L-lysine (PLL). In these binding studies (Fig. 3), PA strains 27853 (type a flagellin), Pa01 (type b flagellin) and 2 additional flagellin type b containing PA strains Ka02 and 25619 (as determined by PCR analysis, data not shown) were immobilized on plates. Additionally, a non-motile PA strain (9721) and thus potentially devoid of a flagellum was also incorporated in the screen as a negative control. LST-007 (0.5 μg/ml) specifically bound strains PAO1, Ka02 and 25619 but was devoid of reactivity towards PA strains 27853 and 9721. Conversely, LST-003 solely reacted with strain 27853. To confirm the specificity of the ELISA, 0.5 μg/ml of a human isotype control mAb failed to react with any of the PA strains (Fig. 3). These data therefore confirm that LST-007 is a highly specific mAb and capable of binding flagellin type b in its natural form as part of intact PA bacteria.

### In vitro biological activity of LST-007 on perturbation of PA motility in soft agar assays

**Figure 4.**
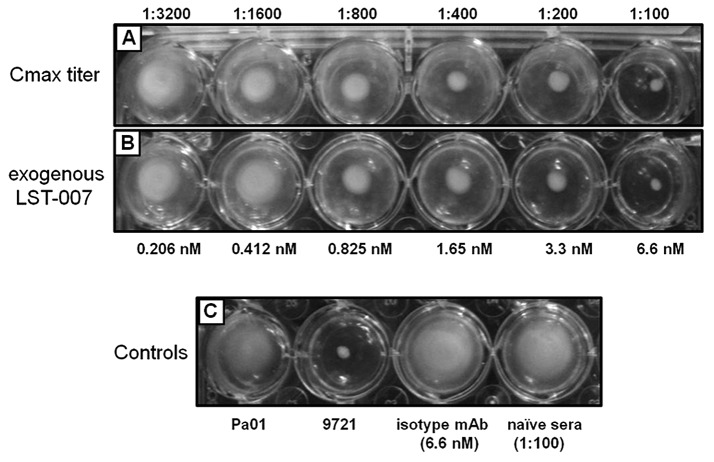
Photomicrographs showing the *de novo* inhibitory effect of LST-007 on PA motility in soft agar assays. Single Pa01 colonies were stabbed into soft agar impregnated with different dilutions of (A) PK-derived pooled Cmax sera (1:3200-1:100) or (B) equivalent calculated amounts of exogenously added LST-007 (0.206-6.6 nM). LST-007 from both sources caused identical, dose-dependent inhibition of bacterial motilities as indicated by the reduced halo effect encircling the origin of bacterial stabbing and growth. (C) No effect on bacterial motility was observed at a 1:100 dilution of naïve mice sera nor 6.6 nM of an irrelevant human isotype mAb. The phenotype of a non-motile bacterial strain 9721 is depicted as well as Pa01 alone.

Based on the proven, high specific reactivity of LST-007 towards PA flagellin type b and the quality of the mAb preparation, bioactivity studies were undertaken by assessing the capability of LST-007 to perturb PA motility in soft-agar impregnated with the mAb. In such studies, LST-007 from disparate sources was employed as follows: i) using Cmax sera from our PK studies whereby sera were diluted from 1:100-1:3,200, and ii) using exogenously added LST-007 from our purified mAb preparation at identical concentrations to the measured serum titer based on the LST-007 Cmax of 804.86 nM (120.67 μg/ml) (Table I). Concentration-dependent inhibition profiles on PA motility was observed with either the *ex vivo* (Fig. 4A) or exogenous source (Fig. 4B) of LST-007 with complete suppression of PA motility observed at 6.6 nM (∼1 μg/ml) mAb.

### PK profile of LST-007 in blood and broncholveolar lavage (BAL) fluid from naïve mice

**Figure 5.**
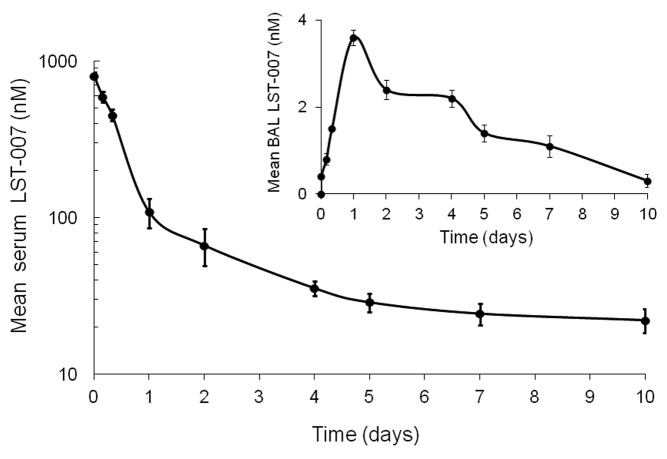
PK profile of LST-007 in sera and BAL fluid from naïve mice. Naïve CD mice were injected i.v. with LST-007 (5 mg/kg) and sacrificed at the various time points shown for simultaneous blood and BAL fluid LST-007 determinations using a highly specific human ELISA. In blood, LST-007 demonstrated a rapid elimination phase during the first 24 h with a much slower phase of elimination up to Day 10. LST-007’s half-life in blood was calculated to be 83.5 h. In contrast to blood, BAL fluid LST-007 concentrations (inset) peaked at 24 h and slowly decreased over the ensuing 9 days.

The PK profile of LST-007 in blood and BAL fluid was investigated following a single i.v. injection of the mAb at a dose of 5 mg/kg in naïve mice. This dose was chosen based on a PK study performed with an anti-RAGE mAb (36) and deemed appropriate by us in terms of our ELISA detection capabilities based on the theoretical concentrations of LST-007 expected in different compartments. In sera, LST-007’s Cmax (at 5 min) was 804.86 nM (120.67 μg/ml) followed by a rapid phase of elimination to a concentration of 111.18 nM (16.67 μg/ml) at 24 h (Fig. 5). A much slower rate of elimination occurred over the next 9 days since the LST-007 concentration was measured at 26.6 nM (4 μg/ml) at Day 10 (Fig. 5). LST-007’s half-life was calculated to be 83.58 h (Table I).

In marked contrast to the blood compartment kinetics, LST-007 exhibited a different PK profile in BAL fluid since mAb concentrations increased over the first 24 h, peaking to 3.6 nM (0.54 μg/ml) at the 24 h time point and decreased slowly over the next 9 days (Fig. 5). LST-007 was still detectable in BAL fluid at Day 10 at a concentration of 0.3 nM (0.045 μg/ml).

### Effect of LST-007 on animal survival in a lethal MDR PA-induced model of pneumonia

**Figure 6.**
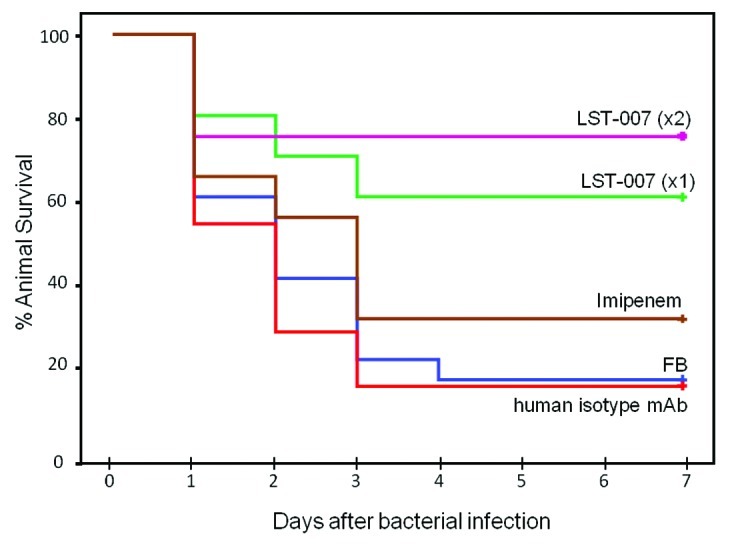
LST-007 markedly improves the survival of mice in a lethal model of MDR PA-induced pneumonia. Mice were infected intra-tracheally with 10^6^ cfu Ka02 (LD_80_) and treated with LST-007 (20 mg/kg) at 60 min post-infection (LST-007x1) in one group (n=20) or with an additional dose +25 h (LST-007x2) in a second group (n=20). Other experimental groups included LST-007 formulation buffer (FB; n=20), a human isotype control mAb (20 mg/kg; n=15) or imipenem (25 mg/kg, bid for 3 days; n=20). Survival was assessed for up to 7 days following infection. By Fisher’s exact test, P=0.0002 for LST-007 (x1) and P<0.0001 for LST-007 (x2) as compared to the FB group. Imipenem treatment was not significant (P=0.1553) vs. the FB group.

**Figure 7.**
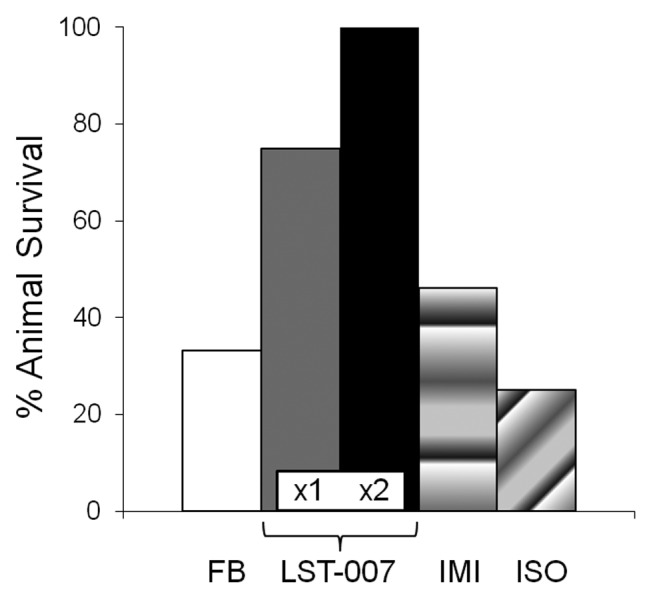
LST-007 (x2) affords complete animal survival between Days 1 and 3 post-infection. The effect of a second i.v. administration of LST-007 (20 mg/kg) given 24 h after infection on survival was investigated. This second dose of LST-007 completely prevented the further decrease in mortality observed with LST-001 (x1) dosing paradigm between 24 and 72 h after infection, allowing 100% survival of mice. LST-007 (x2) effect was specific since two i.v. injections of a human isotype mAb (ISO) at identical dosing to LST-007 failed to attenuate mortality changes during the same period. FB, formulation buffer; IMI, imipenem.

The biological effect of LST-007 on animal survival was investigated in a lethal mouse model of pneumonia driven by an MDR PA strain (Ka02). Initial calibration studies demonstrated that i.t. instillation of 106 cfu Ka02 elicited an LD_80_ at 3 days (data not shown). This mortality profile was used by us in subsequent feasibility studies with LST-007. Following lung infection, mice treated with i.v. FB demonstrated typical lethality curves with 60, 40 and 20% survival at Days 1, 2 and 3, respectively (Fig. 6). In marked contrast, infected animals treated with LST-007x1 or LST-007x2 demonstrated marked improvements in survival at 1–3 days post-infection with 60 and 75% survivors at Day 3 respectively which remained unchanged until Day 7 (Fig. 6). The specificity of this effect was confirmed since an irrelevant human isotype control mAb administered at 20 mg/kg twice at +1 and +25 h, failed to curtail mortality which essentially superimposed on the mortality profile observed with the FB treatment group (Fig. 6). Importantly, imipenem treatment at doses and a route of administration known to be protective against the laboratory strain PAO1 (30,31) was ineffective in preventing Ka02-induced mortality with survival of only 30% at Day 3 (Fig. 6). The importance and significance of a second dosing of LST-007 is underscored in Fig. 7 since the mAb completely abrogated mortality changes noted with LST-007x1 at Day 2 (2/16 deaths) and Day 3 (2/14 deaths).

## Discussion

A call has been heeded by the Infectious Diseases Society of America (IDSA) and the European Medicines Agency (EMA) for the urgent development of novel antibacterial strategies targeting in particular, the ESKAPE group of pathogens (1,37). In the case of *Pseudomonas aeruginosa (*PA), a major dearth exists due to the lack of effective antibacterials targeting MDR PA strains. Currently, most of the anti-PA drugs in late stage pre-clinical testing or early clinical evaluation are modifications of existing drugs, newer combinations of existing drugs or even newer routes of administration of old drugs (38–40). Clearly, innovative remedies are urgently required to combat MDR PA which is a major nosocomial threat, especially in immunocompromised patients.

It is axiomatic that mAbs are proven therapeutics for cancer and immunologic disorders (41), with increasing recognition that mAbs may serve as *bona fide* drugs for a variety of infectious diseases (42,43) including PA (19,20) by targeting surface-expressed virulence factors. We developed and expressed in CHO cells, a fully human mAb (LST-007) against the flagella of PA by targeting flagellin type b, one of the two major protein forms of this appendage. LST-007 was purified to homogeneity from CHO cells, reacted specifically with *E. coli*-expressed flagellin type b and exhibited a high affinity of 7.4x10^−10^ M in SPR assays. These binding profiles were supported by the capability of LST-007 to selectively bind its native target in whole PA bacteria. Since flagellin type b is glycosylated in PA (44) and may have an impact on mAb binding, integrity of LST-007’s recognition towards PA in the fixed ELISA format served as an impetus for bioactivity assays.

Previously, anti-PA flagellin mAbs were reported to inhibit PA motility *in vitro* which presumably laid the basis for their beneficial effects in models of PA infection (27–29). Similarly, LST-007 suppressed PA motility at concentrations close to its K_D_ of the mAbs. Furthermore, we also demonstrated that hyperimmune sera derived from PK studies with LST-007 was similarly bioactive to exogenously added mAb (Fig. 4). This *ex vivo* finding was important for 2 reasons: i) it demonstrated that LST-007 retains bioactivity *in vivo* indicating its likely stability within blood, and ii) it provided us with valuable information regarding LST-007’s target concentration that should allow effective inhibition of PA motility and positive proof-of-concept in our pneumonia model. Indeed, a 1:100 dilution of Cmax sera containing ∼1 μg/ml LST-007 (6.6 nM LST-007) can be regarded as the minimal inhibitory concentration (MIC) that caused complete inhibition of PA motility by comparing its phenotype in soft agar with 9721, the non-motile aflagellated PA strain (Fig. 4).

The PK profile of LST-007 in the blood from naïve mice demonstrated a half-life of ∼84 h, reaching a concentration ∼15% of the Cmax at 24 h during a rapid elimination phase. At such a concentration (16 μg/ml), ∼10X MIC on motility, LST-007 may be expected to be bioactive in a bloodstream PA infection. Nevertheless, LST-007 bioactivity in a pneumonia model is governed by its traversing into the alveolar space from blood. LST-007 concentrations from the same naïve mice were measured in BAL fluid, peaked at 24 h (Cmax, 0.5 μg/ml) and gradually decreased until Day 10 (Fig. 5). Based on an ∼0.5 MIC of LST-007 in BAL fluid at 24 h after dosing with 5 mg/kg, we rationalized that a higher and potential additional dose of LST-007 may be required to allow appropriate pharmacodynamic ‘coverage’ and a desired biological effect in the pneumonia model. When administered a single dose of 20 mg/kg, LST-007 improved survival at each day during the first 3 days during which significant mortality occurred in both control groups. Importantly, a second dose of LST-007, 25 h after infection, completely attenuated mortality changes that occurred at Days 2 and 3. These latter findings would indicate that LST-007 reached the alveolar space at concentrations exceeding 1X MIC which are sustained during the 3 days mortality window. Clinical implications from these findings are 2-fold: i) constant infusion of LST-007 during PA colonization and infection may be the appropriate mode of administration, and ii) LST-007 could be given prophylactically or at least during PA colonization since it traverses into the alveolar space of intact lung. This latter property is presumably due to LST-007 being an IgG1 isotype since the accumulation of the IgM mAb panobacumab in BAL fluid was critically dependent upon the consequences of PA infection (i.e. lung microvascular permeability defects) (17).

A limitation in the present study is that bacterial burden in lungs was not measured. However, since the infecting strain was instilled directly into the lung and there is no evidence that anti-PA mAbs can exert bactericidal effects, reduction in lung bacterial burden are unlikely yet still needs to be verified. Nevertheless, LST-007-mediated suppression of infection throughout the lung or to other organs may occur as reported in the suppression of bacterial dissemination to the spleen in a burn model of PA infection with an anti-type a flagellin mAb (31). Taken together, our data indicated that the beneficial effect of LST-007 on survival may be due to factors that not only target PA motility and accessibility of the bacteria to the TLR5, but also reduced invasiveness into alveolar epithelial cells as well as potential toning down of local inflammatory events driven by monomeric flagellin (23,45). Studies utilizing human alveolar A549 epithelial cells, recombinant flagellins, appropriate infecting PA strains as well as the non-motile 9721 strain, could help to clearly scrutinize the roles of the flagellum in such processes.

Alanine-scanning mutagenesis has shown that the structural requirements for flagellar motility are much more rigid than the permissive nature of TLR5 recognition of flagellin. (23). Interestingly, mouse mAbs raised against PA flagella (27) which impeded motility were shown to bind PA flagellins downstream from amino acid 161 by SDS-PAGE (31). It may be possible that LST-007 similarly targets PA flagellin type b, especially since mutations of a number of conserved C’ terminal amino acid residues in flagellin’s D1 domain resulted in complete abrogation of bacterial motility (23). Appropriate epitope mapping using techniques such as a hybrid β-lactamase display as recently demonstrated for an anti-CD22 immunotoxin CAT-8015 (46), could be very useful to localize LST-007’s site of binding within PA flagellin type b.

From a historical perspective, it is perplexing as to why therapeutic antibodies targeting PA flagellin failed to come to fruition yet reached significant stature in the early 1990’s. Cessation of developmental efforts were probably swayed by the disappointing clinical studies with anti-sepsis agents including mAbs (47,48). Additionally, the need to develop two separate mAbs targeting flagellin type a and b for complete anti-PA coverage may have been an issue, especially during a period where many of the methodological aspects concerning the development of therapeutic mAbs were still in their infancy. Today, such issues can be overcome, as exemplified by the prior screening of infected PA patients to ensure compatibility for immunotherapy (19), streamlined mAb manufacturing approaches (14) or even potential design of bispecific mAbs (49). Flagellin represents a viable target for therapeutic intervention which is further fueled by two additional findings. In translational studies, whereby polymorphisms in the TLR5 and subsequent flagellin hyporesponsiveness are associated with improved health indicators in Crohn’s disease (50), CF (51) and systemic lupus erythematosus (52). Secondly, the highly encouraging clinical data in CF patients using oral chicken IgY (53) whereby PA flagellin was proven to be the major immunoreactive antigen bound by this polyclonal therapeutic from proteomic studies (54).

In summary, LST-007 represents a *bona fide* fully human IgG1 mAb targeting PA flagellin type b which was shown to afford significant improvement in survival against an MDR PA-induced pneumonia, superceding standard care of the treatment. In an era of limited therapeutics targeting MDR PA, neutralizing anti-PA mAbs may not only provide a new monotherapeutic strategy but could additionally synergize with existing antibiotics. Such a desired effect could shepherd these precious drugs from resistance mechanisms. Our current data provide us with significant momentum in continuing the development of LST-007 with the intent of evaluating its clinical efficacy. Such patients could include those within the critical care setting (e.g. ventilator-associated pneumonia) or CF patients. Alternate indications, especially those where PA may be the sole infectious culprit (e.g. urinary tract infections, necrotizing malignant external otitis), could also represent highly feasible patient populations to demonstrate clinical proof-of-concept.

## Figures and Tables

**Table I.** Pharmacokinetic analysis of LST-007 in naïve mice blood following the i.v. injection of a single dose of 5 mg/kg.

LST-007 PK analysis			
AUC	(0 to 240)	mvc/ml * h	2463.34
	(0 to infinity)	mcg/ml * h	2772.88
Tmax	(0 to 240)	h	0.083
Cmax	(0 to 240)	mcg/ml	120.67
Cmin	(0 to 240)	mcg/ml	2
Cavg	(0 to 240)	mcg/ml	10.26
	Ke	1/h	0.009565
	T1/2	h	83.58
